# Four Dairy Products Mitigates Sarcopenia in Mice by Modulating Muscle Inflammation, Autophagy, and Protein Degradation

**DOI:** 10.1002/fsn3.70540

**Published:** 2025-07-14

**Authors:** Meng Sun, Tong Wu, Ruoyu Wang, Yuxin Ma, Yaxin Han, Yanmei Hou, Zhaofeng Zhang

**Affiliations:** ^1^ Department of Nutrition and Food Hygiene, School of Public Health Peking University Beijing China; ^2^ Hyproca Nutrition Co., Ltd Changsha City Hunan Province China; ^3^ Beijing's Key Laboratory of Food Safety Toxicology Research and Evaluation Beijing China

**Keywords:** bovine milk, goat milk, gut microbiota, sarcopenia

## Abstract

Sarcopenia, characterized by progressive loss of muscle mass and strength, poses a significant public health challenge. However, the specific role of dairy products in preventing sarcopenia is not well understood. This study investigated the effects of different dairy products on muscle metabolism, focusing on the differences between goat and bovine milk, the impact of dairy fat content, and the potential synergistic effects of vitamin D and calcium. Sixty male C57BL/6 mice (8 months old) were randomly divided into six groups and orally administered with 7 g/kg/day of goat whole milk (GWM), goat low‐fat milk (GLM), goat fortified vitamin D and calcium low‐fat milk (GFM), or bovine whole milk (BWM), respectively. Sarcopenia was induced using intraperitoneal dexamethasone injections (5 mg/kg) for 8 weeks. Grip strength, bone mineral density (BMD), fat and lean weight, muscle morphology, autophagy, inflammation, host metabolism, and gut microbiota were assessed. Sarcopenic mice exhibited decreased lean weight and grip strength. While all dairy products increased lean weight, they did not affect grip strength significantly. At the molecular level, all dairy products activated the PI3K/Akt/mTOR pathway, reduced AMPK phosphorylation, and enhanced muscle regeneration, with GFM being most effective in upregulating MyoG expression. Dairy intake also promoted autophagy by increasing LC3B expression and reducing p62 levels while significantly lowering inflammation markers, including CRP, IL‐1β, IL‐6, and TNF‐α. Gut microbiota analysis revealed that all dairy interventions enriched beneficial genera, with *Leuconostoc* present in all groups and *Acinetobacter* and *Lactococcus* enriched in goat milk groups. Notably, GLM and GFM increased *Staphylococcus sciuri* , which may contribute to muscle health benefits. These findings suggest that dairy consumption, particularly GFM, improves muscle mass, regulates autophagy, reduces inflammation, and modulates gut microbiota composition, providing experimental evidence for sarcopenia prevention and management.

## Graphical abstract

Dairy consumption, particularly goat fortified vitamin D and calcium low‐fat milk, could improve muscle mass and reduce fat weight in sarcopenic mice. Dairy consumption has the potential to improve muscle mass and prevent sarcopenia by modulating autophagy, reducing inflammation, and regulating gut microbiota. The interaction between dairy intake, gut microbiota, and circulating metabolites indicates a potential mechanism for the beneficial effects of dairy in preventing sarcopenia.

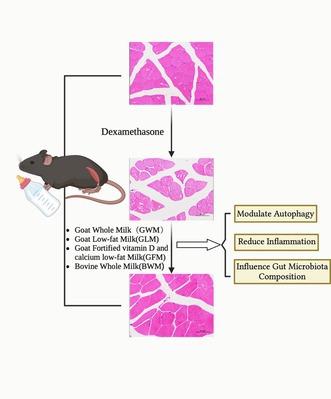

## 1 Introduction

Sarcopenia is a condition characterized by a progressive loss of muscle mass and strength (Cruz‐Jentoft and Sayer 2019), emerging as an important public health issue. A meta‐analysis of 151 studies showed that the global prevalence of sarcopenia varies from 10% to 27% (Petermann‐Rocha et al. 2022). As the country holding the largest elderly population (Chen, Giles, et al. 2022), the prevalence of sarcopenia in China is 12.9% in men and 11.2% in women for community‐dwelling older adults, and 29.7% in men and 23.0% in women for hospitalized older adults (Chen et al. 2021). An increasing number of studies have shown an association between sarcopenia and multiple adverse health consequences, including frailty, falls, disability, and mortality (Norman and Otten 2019). Sarcopenia seriously affects the quality of life of the elderly and brings a huge health and socioeconomic burden, hindering healthy aging. Therefore, early targeted interventions should be taken to delay or even reverse the progression of sarcopenia.

In the process of aging, muscle synthesis decreases and muscle degradation increases, leading to sarcopenia, and inflamm‐aging is a possible trigger. Moreover, malnutrition in the elderly exacerbates the occurrence of sarcopenia. Previous studies have found that changes in important cell signaling pathways affect muscle synthesis and degradation. For example, activation of the phosphatidylinositide 3‐kinases (PI3K)/protein kinase B (Akt) pathway leads to increased protein synthesis and muscle hypertrophy and prevents protein degradation in skeletal muscle (Wiedmer et al. 2021). Genes involved in myogenic differentiation, such as myogenic differentiation (MyoD) and myogenin (MyoG), compromise muscle regenerative capacity (Braun and Marks 2015). Additionally, the imbalance of intestinal flora during aging can lead to inflammatory aging and further sarcopenia (Ticinesi et al. 2019).

Dietary interventions have gained considerable attention as a non‐pharmacological approach to mitigating sarcopenia. Dairy products, as a rich source of high‐quality protein, have been shown to positively influence muscle health (Cuesta‐Triana et al. 2019). The Asian Working Group for Sarcopenia recommended dairy consumption for sarcopenic individuals to enhance muscle mass and function (Chen, Arai, et al. 2022). Despite these recognized benefits, it remains unclear whether different types of dairy products exert distinct effects on muscle metabolism. Goat milk reportedly contains higher levels of bioactive compounds—including oligosaccharides, conjugated linoleic acid (CLA), short‐chain fatty acids, monounsaturated and polyunsaturated fatty acids, and phytoestrogens—which are associated with anti‐inflammatory and metabolic benefits (Turck 2013). Furthermore, the smaller and more uniform fat globules and protein particles in goat milk, compared to bovine milk, may enhance its digestibility and bioavailability (Collard and McCormick 2021). These compositional differences raise important questions about their potential to translate into distinct physiological effects on muscle metabolism.

Building on this foundation, another underexplored aspect is the role of dairy fat content in modulating muscle health, particularly in the context of osteosarcopenic obesity—a subtype of sarcopenia characterized by both muscle loss and increased adiposity (Conforto et al. 2024). On one hand, dairy fat supplies essential fatty acids and bioactive lipids, such as CLA and medium‐chain fatty acids (MCFAs), which may contribute to anti‐inflammatory responses and metabolic regulation (Gómez‐Cortés et al. 2018). On the other hand, excessive fat intake could worsen metabolic dysregulation and further promote obesity‐related muscle deterioration (Ilich et al. 2020). This dichotomy underscores the need to understand whether high‐fat versus low‐fat dairy products differ in their impact on muscle metabolism and sarcopenia outcomes, thereby informing more targeted nutritional strategies.

In addition, while vitamin D and calcium are individually well recognized for their contributions to bone and muscle health, their potential synergistic effects remain largely unexplored. Although numerous studies have focused on their individual roles (Santiago et al. 2021; Remelli et al. 2019; Terrell et al. 2023), few have addressed whether their combined supplementation could confer additional benefits. Integrating these insights, it becomes imperative to determine if fortifying dairy products with vitamin D and calcium might further potentiate the beneficial effects on muscle health, thereby addressing a key gap in current nutritional strategies for sarcopenia prevention.

Therefore, to further explore the effects of dairy products on gut microbiota composition, host metabolism, inflammation, autophagy, and muscle degradation in individuals with sarcopenia, this study established an animal model of sarcopenia. By integrating metabolomics and 16S rRNA gene sequencing technologies, we aim to systematically investigate the ameliorative effects of different types of dairy products on sarcopenia and their potential mechanisms, providing theoretical support and practical evidence for precise nutritional interventions in sarcopenia.

To address these gaps and provide experimental evidence for targeted nutritional interventions in sarcopenia, we developed a sarcopenia mouse model and investigated the effects of dairy consumption on muscle metabolism. By integrating metabolomics and 16S rRNA sequencing, our study elucidates the interplay between dairy intake, muscle signaling pathways, and gut microbiota, thereby informing precise dietary strategies for sarcopenia prevention and management.

## 2 Material and Methods

### 2.1 Materials and Reagents

Common feedstuffs according to the national standard of China for laboratory mouse feed (GB 14924.3) were obtained from Beijing Keaoxieli Feed Co. Ltd. Dexamethasone was purchased from Beijing MREDA Technology Co. Ltd., milk powder from Hyproca Nutrition Co. Ltd., atropine sulfate monohydrate from Shanghai Macklin Biochemical Co. Ltd., and Zoletil 50 from France LDBIO Diagnostics Co. Ltd. Primary antibodies against PI3K, Akt, mammalian target of rapamycin (mTOR), adenosine 5′‐monophosphate‐activated protein kinase (AMPK), p‐PI3K, p‐Akt, p‐mTOR, p‐AMPK, LC3B, and Beclin1 were used from CST Biological Reagents Co. Ltd., primary antibody against MyoD1 from ABclonal Biotech Co. Ltd., and primary antibodies against MyoG, p62, and β‐actin and secondary antibodies from Abcam Co. Ltd.

### 2.2 Animal Treatment

Sixty male SPF C57BL/6 mice (8 months old) were obtained from Beijing Weitong Lihua Experimental Animal Technology Co. Ltd. and acclimatized for 7 days with a standard diet and free access to drinking water. The study protocol was approved by the Biomedical Research Ethics Committee of Peking University (LA2022011) and followed the Regulations for the Administration of Affairs Concerning Experimental Animals.

The mice were randomly assigned to six groups (*n* = 10 each): normal control (NC), sarcopenia (SOP), goat whole milk (GWM), goat low‐fat milk (GLM), goat fortified vitamin D and calcium low‐fat milk (GFM), and bovine whole milk (BWM). The NC group received 5 mg/kg saline intraperitoneally, while the other groups received dexamethasone. The four intervention groups received 7 g/kg of their respective milk type orally. The experiment lasted 8 weeks. The intervention dosage criteria for milk powder was 54 g/d for a person weighing 70 kg. The mouse dose was equivalent to 9.2 times the human dose, calculated according to the body surface area conversion factor. Body weight, food intake, and water intake were monitored throughout the experiment. The detailed animal experimental design was shown in Figure S1.

### 2.3 Muscle Strength and Mass Determination

Forelimb grip strength was measured using a grip strength meter (Beijing Zhongshi Dichuang Technology Development Co. Ltd) and the average of three trials was recorded. The mice were then anesthetized with atropine sulfate (0.1 mg/kg) and Zoletil 50 (25 mg/kg) intramuscularly and scanned with dual‐energy X‐ray absorption (DXA) (Lexxos‐2000; Medlink, France) to assess bone mineral density (BMD), fat, and lean weight. After euthanasia, the bilateral gastrocnemius muscles and intraperitoneal fat were collected and weighed.

### 2.4 Morphology of Gastrocnemius Muscle

Gastrocnemius muscle was fixed in 4% paraformaldehyde at 4°C, embedded in paraffin, sectioned, and stained with HE following routine practices (Doornebal et al. 2013). Images were obtained with a light microscope (Olympus BX43).

### 2.5 Muscle Regeneration, Autophagy, Inflammation, and Biomarkers of Aging Determination

The activation states of the PI3K/Akt/mTOR and AMPK pathways, the expression levels of muscle regeneration indices MyoD1 and MyoG, and the autophagy proteins LC3B, p62, and Beclin1 in the gastrocnemius muscle were analyzed by western blotting as described previously (Wang et al. 2023). Protein bands were quantified with ImageJ software. CRP, inflammatory cytokines (IL‐1β, IL‐6, and TNF‐α), aging biomarkers CXCL10, CX3CL1, and 8‐hydroxyguanosine (8‐oxo‐Gsn) in the gastrocnemius muscle were measured with mouse ELISA kits following the manufacturer's protocol.

### 2.6 Untargeted Metabolomics Profiling Based on GC‐TOF/MS

The XploreMET platform (Metabo‐Profile, Shanghai, China) was used for untargeted metabolomics profiling. The serum sample preparation procedures followed previously published methods with minor modifications (Qiu et al. 2014). A robotic multipurpose sample with dual heads (Gerstel, Muehlheim, Germany) performed sample derivatization and GC‐TOF/MS analysis.

### 2.7 16S rRNA Gene Amplicon Sequencing

Microbial DNA extraction and 16S rRNA gene sequencing The CTAB method was used to extract microbial DNA from the cecal contents of the mice. The V3‐V4 region of the 16S rRNA gene was amplified with the forward primer 341F (CCTAYGGGRBGCASCAG) and the reverse primer 806R (GGACTACHVGGGTWTCTAAT) following the PCR program described previously (Peng et al. 2019). PCR products were detected by 2% agarose gel electrophoresis; according to the determined concentration, the samples were mixed for isoconcentration, and then the PCR products were purified by electrophoresis with 1 × TAE buffer and 2% agarose gel. NEB Next Ultra DNA Library Prep Kit for Illumina was used to construct the libraries. The library quality was assessed by Agilent 5400 and Q‐PCR. The qualified libraries were sequenced using an Illumina NovaSeq 6000 Sequencer.

### 2.8 Statistical Analysis

The experimental data are expressed as means and standard deviation ($\bar{X}$ ± SD). Homogeneity of variance and normality tests were performed before data analysis. ANOVA was used for multigroup comparison followed by the least significant difference (LSD) test for pairwise comparison. A *p* value of 0.05 was considered significant.

We used linear discriminant analysis effect size (LEfSe) analysis to compare genera and species of gut microbiota with significant differences among groups, applying a false discovery rate (FDR) of 0.05 for the Kruskal–Wallis test and an LDA score higher than 2.0. We then predicted the function of gut microbiota with PICRUSt2 and KEGG pathway library. Based on previous research (Shuai et al. 2021), we generated a dairy‐microbial score as a new gut microbial feature to represent the gut bacteria group associated with different types of dairy and examined the differences in the scores among groups. We also performed Spearman correlation analysis between the dairy‐microbial score, differential bacteria, and phenotype data.

We performed orthogonal partial least square discriminant analysis (OPLS‐DA) and univariate analyses (Student's *t* test and *U* test) to explore potential metabolic biomarkers, using a variable influence on projection (VIP) of 1.5 and a *p* value of 0.05 for statistical significance. We also performed Spearman correlation analysis between the dairy‐microbial score, phenotypes, metabolic biomarkers, and predicted metabolic function–derived bacterial data. We used R 4.2.0 (R Core Team) for all analyses.

## 3 Results

### 3.1 Effect of Four Dairy Products on the Weight in Sarcopenic Mice

**FIGURE 1:**
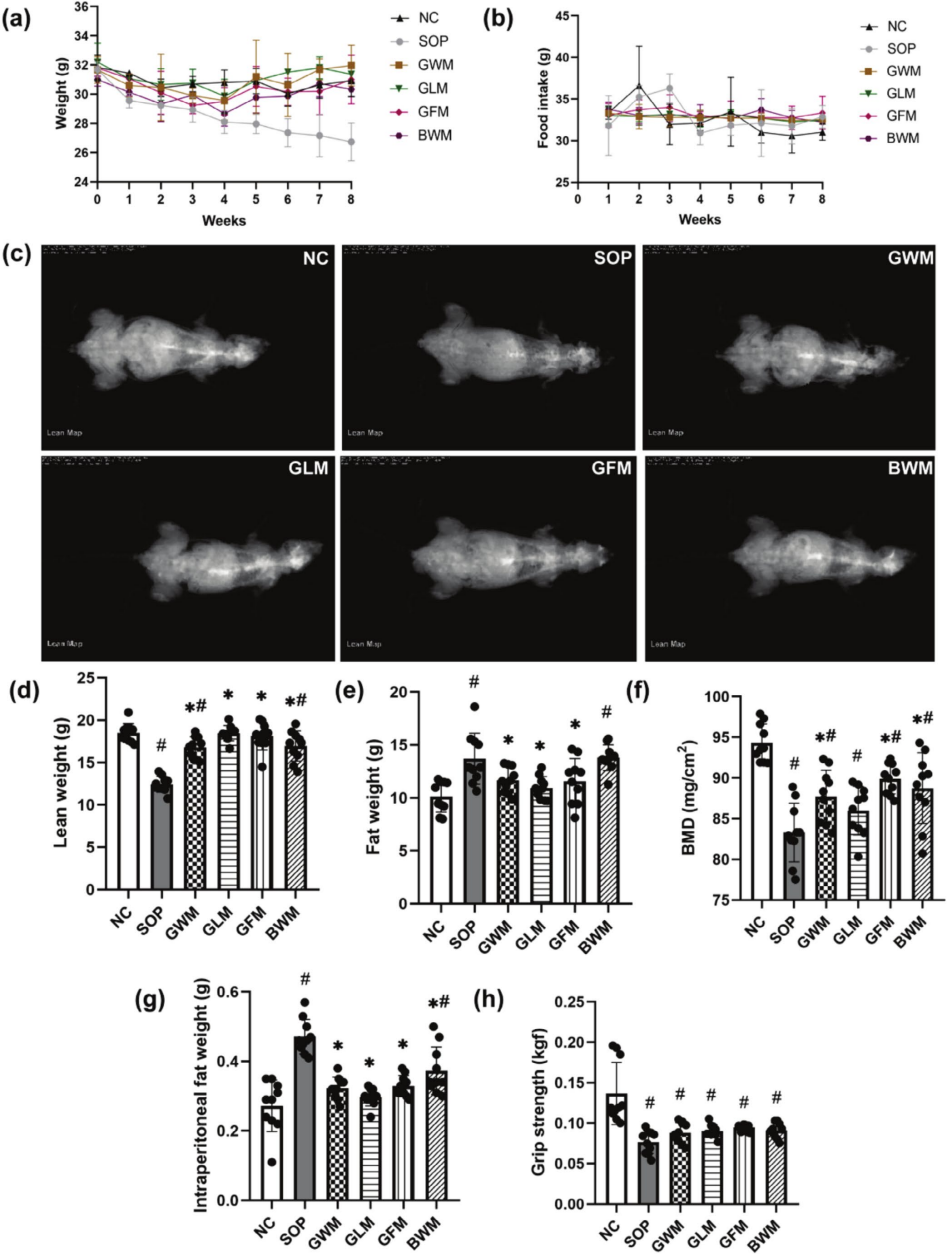
Weekly body weight and food intake, scanning image, bone mineral density (BMD), fat and lean weight by dual‐energy X‐ray absorption (DXA) scan analyses, and wet weight of intraperitoneal fat and forelimb grip strength of sarcopenic mice treated for 8 weeks with goat and bovine milk (*n* = 10). (a) Body weight. (b) Food intake. (c) Scanning image for lean weight. (d) Lean weight. (e) Fat weight. (f) BMD. (g) Wet weight of intraperitoneal fat. (h) Forelimb grip strength. ^#^ indicates significant difference compared with the NC group (*p* < 0.05); * indicates significant difference compared to the SOP group (*p* < 0.05).

During the intervention period, the weight of mice in the SOP group continuously decreased while it was maintained in the NC group (Figure 1a). After the intervention, the trend of weight loss was reversed in all four groups. No significant difference in food intake was observed among all groups over time (Figure 1b; *p =* 0.90).

### 3.2 Effect of Four Dairy Products on the Muscle Mass and Strength in Sarcopenic Mice

Sarcopenic mice had decreased lean weight and BMD and increased fat weight based on DXA scans (Figure 1c–f; *p* < 0.05). Four dairy products could significantly mitigate the decrease in lean weight (*p < 0*.05), especially GLM and GFM interventions, which could achieve similar levels of lean weight to the NC group. Three types of goat milk effectively decreased fat weight (*p* < 0.05), whereas bovine whole milk failed. GWM, GFM, and BWM significantly improved BMD (*p* < 0.05). The wet weight of intraperitoneal fat in the SOP mice prominently increased but was decreased by four dairy products based on weighing (Figure 1g; *p* < 0.05).

The forelimb grip strength of the SOP mice prominently decreased based on the grip strength test (Figure 1h; *p* < 0.05) and modestly increased in all intervention groups, although this effect did not reach statistical significance.

### 3.3 Effect of Four Dairy Products on Pathological Changes in Gastrocnemius Muscle in Sarcopenic Mice

**FIGURE 2:**
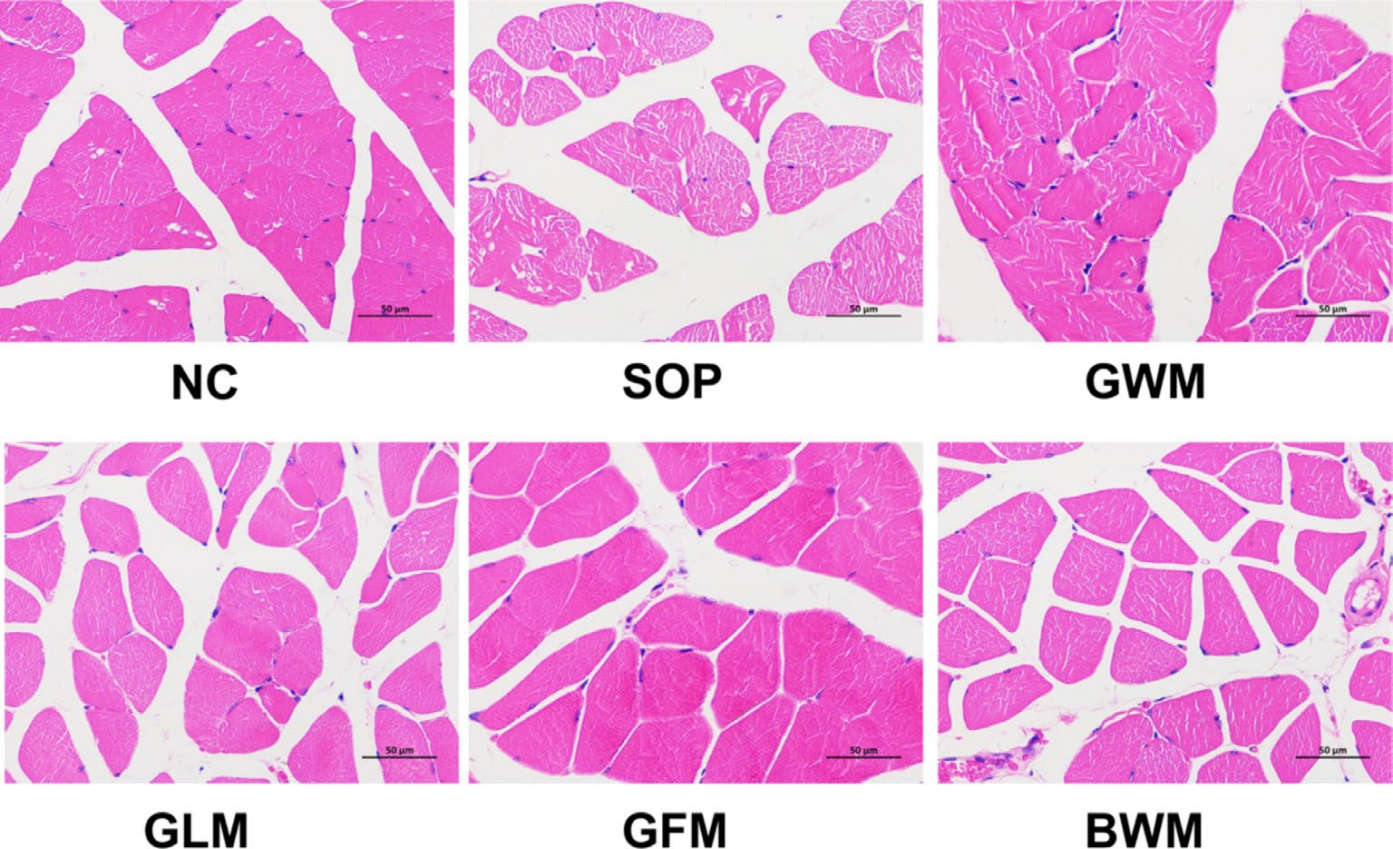
Light microscopy of the gastrocnemius muscle stained by hematoxylin and eosin (HE) of sarcopenic mice treated for 8 weeks with goat and bovine milk. Scale bar: 50 μm; original magnification 400 × .

Figure 2 shows the gastrocnemius muscle stained by HE in different groups. The muscle fiber cells of the NC group mice were neatly arranged and the same in size. In contrast, the SOP group showed unevenly distributed muscle cells, a higher number of nuclei, and increased gaps between muscle fibers. GWM and GLM intervention restored the neat arrangement of muscle cells, yet muscle fibers were partially loosely packed. GFM improved muscle fiber arrangement and decreased muscle fiber atrophy. Muscle fibers in the BWM group were clearly formed, yet they were small and loosely packed.

### 3.4 Four Dairy Products Modulated Muscle Regeneration and Autophagy by Regulating Different Protein Targets in Sarcopenic Mice

**FIGURE 3:**
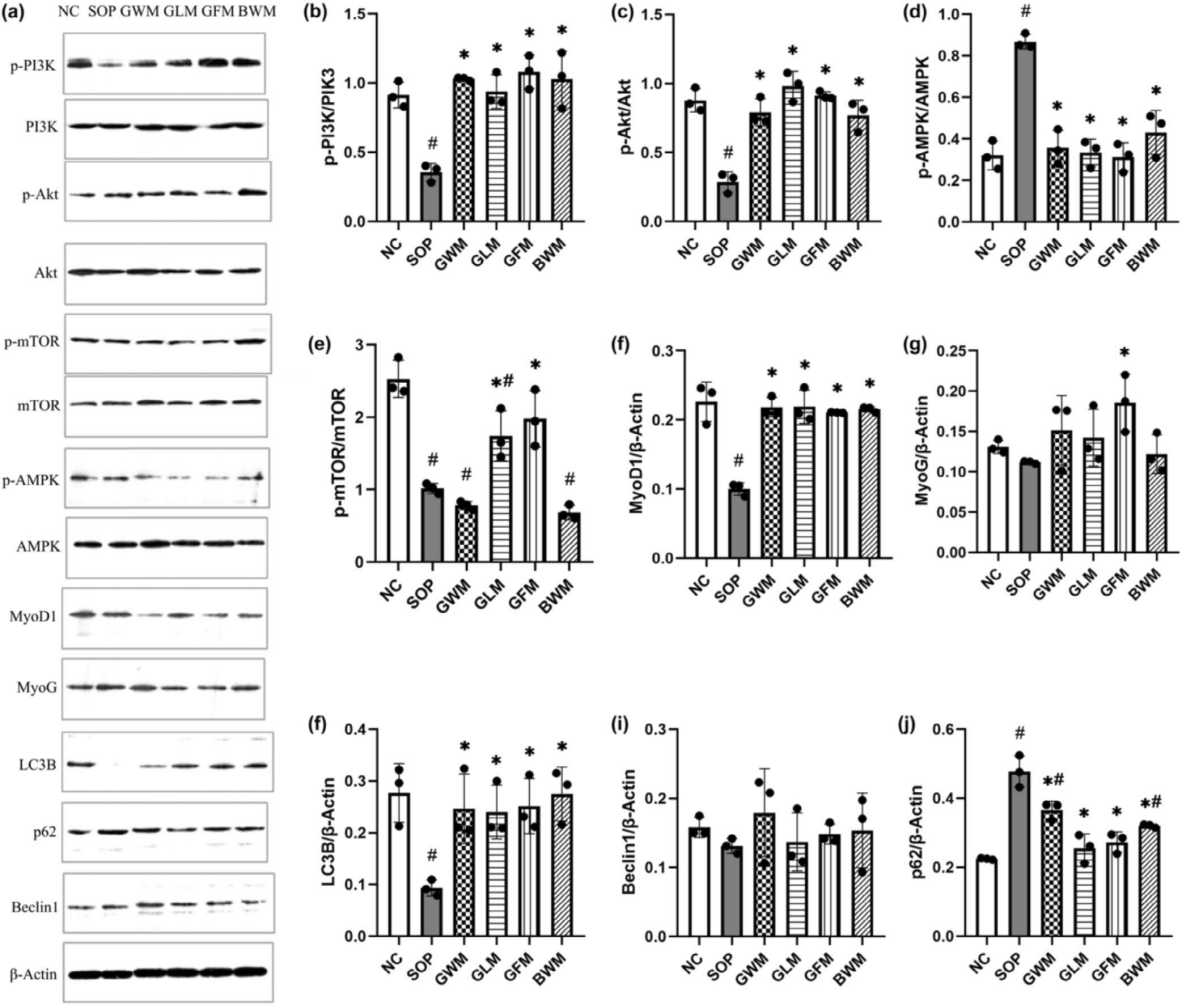
Western blot of PI3K, Akt, AMPK, mTOR, p‐PI3K, p‐Akt, p‐AMPK, p‐mTOR, MyoD1, MyoG, LC3B, p62, and Beclin1 protein levels in sarcopenic mice treated for 8 weeks with goat and bovine milk. (a) Western blot images. (b–j) Quantification of Western blot bands. ^#^ indicates significant difference compared with the NC group (*p* < 0.05); * indicates significant difference compared to the SOP group (*p* < 0.05).

The expression levels of total PI3K, Akt, AMPK, and mTOR in the gastrocnemius muscle were similar among groups (Figure 3a). However, the phosphorylation levels of these proteins showed significant differences. The SOP group had lower phosphorylation of PI3K, Akt, and mTOR and higher phosphorylation of AMPK than the NC group (Figure 3b–e). Four dairy products increased PI3K and Akt phosphorylation and decreased AMPK phosphorylation, while goat low‐fat milk and goat fortified low‐fat milk also enhanced mTOR phosphorylation (*p* < 0.05). The SOP group also had lower expression levels of MyoD1 and MyoG, which are muscle‐specific transcription factors. MyoD1 expression was restored by all four dairy products and MyoG expression was increased only by goat fortified low‐fat milk (Figure 3f,g; *p* < 0.05). In addition, the SOP group had lower expression of LC3B and higher expression of p62, which are markers of autophagy. Four dairy products reversed these changes (Figure 3h–j; *p* < 0.05). The expression level of Beclin1, another autophagy‐related protein, was not affected by any treatment.

### 3.5 Effect of Four Dairy Products on Inflammation and Biomarkers of Aging in Sarcopenic Mice

Table 1 shows the expression levels of inflammatory and oxidative stress markers. The SOP group had significantly higher expression of CRP, IL‐1β, IL‐6, and TNF‐α than the NC group (*p <* 0.05). These four indicators were significantly reduced by all four dairy products (*p <* 0.05). CXCL10 expression was also decreased by four dairy products compared to the SOP group (*p* < 0.05), while CX3CL1 and 8‐oxo‐Gsn expression did not differ among groups.

**TABLE 1:** Inflammatory cytokines and chemokines in sarcopenic mice treated for 8 weeks with goat and bovine milk (*n* = 10).

Groups	NC	SOP	GWM	GLM	GFM	BWM
CRP (ng/mg)	20.78 ± 1.27	38.12 ± 1.04^a^	25.83 ± 0.77^a^, ^b^	27.65 ± 0.93^a^, ^b^	25.62 ± 1.30^a^, ^b^	26.07 ± 0.90^a^, ^b^
IL‐1β (pg/mg)	18.92 ± 2.65	24.13 ± 1.56^a^	16.69 ± 0.85^b^	15.98 ± 1.76^b^	13.78 ± 0.85^a^, ^b^	13.81 ± 0.82^a^, ^b^
IL‐6 (pg/mg)	15.92 ± 0.77	18.76 ± 1.38^a^	14.29 ± 0.77^b^	14.57 ± 1.26^b^	15.13 ± 0.91^b^	11.40 ± 0.27^a^, ^b^
TNF‐α (pg/mg)	47.93 ± 3.01	65.91 ± 4.23^a^	44.53 ± 0.84^b^	45.62 ± 0.76^b^	45.56 ± 0.88^b^	43.37 ± 0.97^b^
CXCL10 (pg/mg)	77.48 ± 4.86	110.53 ± 17.01^a^	44.81 ± 4.24^a^, ^b^	39.38 ± 13.57^a^, ^b^	35.42 ± 1.43^a^, ^b^	35.67 ± 1.01^a^, ^b^
CX3CL1 (ng/mg)	0.84 ± 0.08	0.99 ± 0.11	0.85 ± 0.08	0.81 ± 0.17	0.74 ± 0.03	0.71 ± 0.01
8‐oxo‐Gsn (ng/mg)	0.31 ± 0.04	0.36 ± 0.52	0.30 ± 0.06	0.30 ± 0.04	0.29 ± 0.03	0.34 ± 0.02

*Note:* Values are expressed as the mean ± SD.

^a^ Indicates significant difference compared with the NC group (*p* < 0.05).

^b^ Indicates significant difference compared to the SOP group (*p* < 0.05).

### 3.6 Four Dairy Products Modulated Distinct Gut Microbiota and Metabolites in Sarcopenic Mice

Multiple genera and species were enriched in the four intervention groups, respectively (Figure S2; Tables S1 and S2). Among those genera, Leuconostoc was enriched in all dairy groups. Moreover, Acinetobacter and Lactococcus were enriched in all goat dairy groups. At the species level, *Acinetobacter guillouiae* was enriched in all goat dairy groups. Additionally, *Staphylococcus sciuri* was identified as a biomarker of goat low‐fat milk intake and fortified vitamin D and calcium low‐fat milk intake.

**FIGURE 4:**
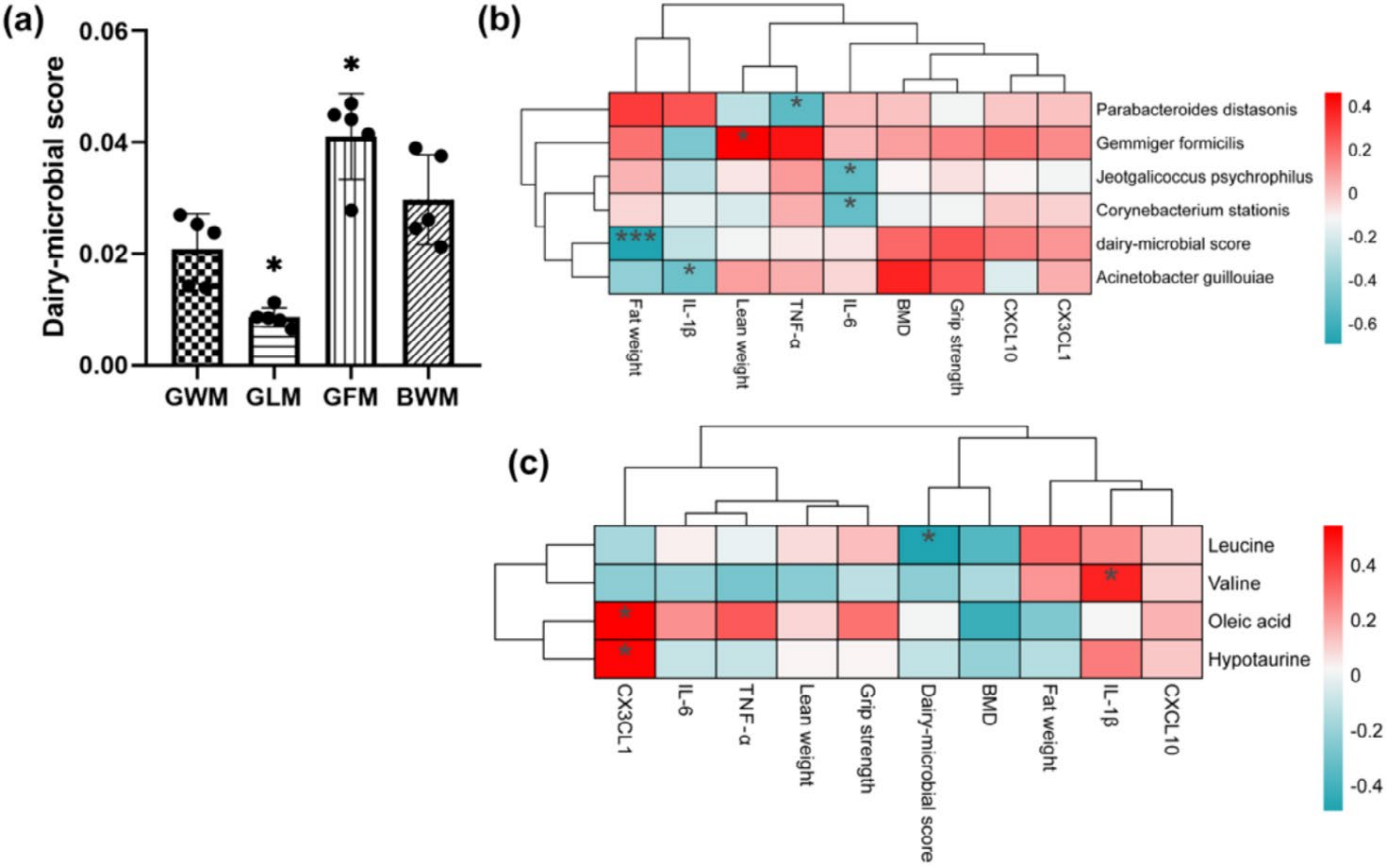
Phenotypic data, dairy‐microbial score, gut microbiota, and metabolic biomarkers in sarcopenic mice treated for 8 weeks with goat and bovine milk. (a) Dairy‐microbial score. * indicates significant difference compared to the GWM group (*p* < 0.05). (b) Heatmap of the correlation coefficients between gut microbiota, dairy‐microbial score and phenotypic data. (c) Heatmap of the correlation coefficients between metabolic biomarkers, dairy‐microbial score and phenotypic data. The coloring indicates the direction of association (red: positive; blue: negative). *p value< 0.05; **p value< 0.01; ***p value< 0.001.

We observed a statistically significant difference in dairy‐microbial score generated from bacteria biomarkers in the GLM and GFM groups compared to the GWM group (Figure 4a; *p* < 0.05). The correlation analysis (Figure 4b) indicated that dairy‐microbial score was negatively related to the fat weight level. IL‐1β was negatively related to *Acinetobacter guillouiae* , IL‐6 was negatively related to *Corynebacterium stationis* and *Jeotgalicoccus psychrophilus* , and TNF‐α was negatively related to *Parabacteroides distasonis* (*p* < 0.05, *r* < −0.4). Lean weight was positively related to *Gemmiger formicilis* (*p* < 0.05, *r* > 0.4).

The metabolic biomarkers in each group are shown in Table S3. The correlation analysis (Figure 4c) indicated that the dairy‐microbial score was negatively associated with leucine (*p <* 0.05, *r* < −0.4). IL‐1β was positively related to valine (*p* < 0.05, *r* > 0.4). CX3CL1 was positively related to oleic acid and hypotaurine. The heatmap in Figure S3 showed the correlation between metabolite types and gut microbiome functional pathways. The functions of intestinal flora closely related to host metabolism include immune disease, metabolism of terpenoids and polyketides, and signaling molecules and interaction.

## 4 Discussion

This study utilized a sarcopenia mouse model to systematically evaluate the effects of different dairy products on the improvement of sarcopenia, and revealed their potential benefits at the levels of body composition, molecular mechanisms, and omics. Overall, all dairy interventions were able to improve lean body mass and bone mineral density, reduce fat mass, and exert effects at the molecular level through activation of the PI3K/Akt/mTOR pathway, reduction of AMPK phosphorylation, enhancement of autophagy, and inhibition of inflammatory factors. Meanwhile, certain differences were observed between the groups in some indicators, suggesting that the source of milk, fat content, and fortification with vitamin D and calcium may have different mechanisms of action in improving sarcopenia, providing experimental evidence for the application of dairy products in preventing and controlling sarcopenia.

Firstly, regarding the effects of different milk sources, we found that while cow's milk intervention also improved body composition, goat's milk, particularly the low‐fat formula, showed more pronounced benefits. Compared to the BWM group, mice in the GLM and GFM groups showed higher lean body mass and BMD in DXA analysis, with a greater reduction in inflammatory factors (CRP, IL‐1β, IL‐6, and TNF‐α). This result may be attributed to higher levels of oligosaccharides, CLA, short‐chain fatty acids, and other bioactive components in goat's milk, along with its smaller and more uniform fat globules, which improve the bioavailability of its nutrients. These data support the potential unique advantages of goat's milk in improving muscle metabolism and inflammation, which is consistent with existing literature (Petrella et al. 2024).

Next, our study found that dairy products with different fat contents exhibited differences in the regulation of the PI3K/Akt/mTOR signaling pathway and autophagy. Compared to GWM, GLM significantly reduced p62 expression, indicating that GLM may be more advantageous for promoting autophagic flux. Autophagy is an essential mechanism for maintaining muscle homeostasis, and its disruption can lead to the accumulation of abnormal proteins and damaged organelles, exacerbating protein degradation and accelerating the progression of sarcopenia (Paez et al. 2023). In addition to autophagy regulation, changes in dairy fat content may also influence muscle protein synthesis through the PI3K/Akt/mTOR pathway. This study found that GLM activated downstream mTOR more significantly. mTOR is a key regulator of protein synthesis, and its activation promotes muscle growth and inhibits protein degradation (Yoon 2017), suggesting that low‐fat goat's milk has a greater advantage in enhancing protein synthesis and maintaining muscle mass. This difference may be related to the complex effects of high‐fat dairy products on metabolic regulation, where excessive fat intake could alter energy homeostasis, thereby affecting downstream mTOR signaling.

Obesity‐related sarcopenia (osteosarcopenic obesity, OSO) is a complex phenotype characterized by osteoporosis, muscle loss, and fat accumulation (Ilich et al. 2014). Its development is closely associated with metabolic disorders and is considered to be independently related to muscle function decline and increased mortality risk (Reinders et al. 2016). Bioactive lipids in dairy fat, such as conjugated linoleic acid and medium‐chain fatty acids, may offer protective effects by regulating metabolic signaling pathways, but increased fat content may impact the PI3K/Akt/mTOR pathway and autophagic activity, thereby influencing muscle homeostasis. The results of this study suggest that in nutritional interventions for obesity‐related sarcopenia, controlling fat content is more crucial than the type of lipids. Moreover, MFGM, an important component of dairy products, may play a role in how fat content affects muscle metabolism. Supplementation with MFGM in animals showed lower LC3B levels, and MFGM may regulate autophagic activity (Yu et al. 2022). However, its specific mechanism requires further research.

The importance of vitamin D and calcium supplementation in bone health has been widely studied, but their synergistic effects in the prevention and control of sarcopenia are not yet fully clarified. This study found that the low‐fat goat's milk group fortified with vitamin D and calcium (GFM) showed superior effects in improving bone mineral density, promoting muscle regeneration, and inhibiting inflammation. Specifically, this group showed a significant improvement in BMD, and both MyoD1 and MyoG expression were significantly increased, while other dairy intervention groups mainly restored only MyoD1 expression. MyoD family transcription factors consist of a group of related basic helix–loop–helix DNA‐binding proteins (Buckingham and Rigby 2014). MyoG is the only muscle regulatory factor gene that is expressed in all skeletal muscle cell types (Hasty et al. 1993). MyoD1 is a transcriptional activator that promotes the expression of muscle‐specific genes and works together with MyoG to regulate muscle differentiation (Akizawa et al. 2013). Therefore, the GFM group may play a more significant role in promoting muscle differentiation through activation of the MyoG signaling pathway. Additionally, this group exhibited a more pronounced anti‐inflammatory effect, which may be related to vitamin D's role in enhancing calcium absorption and modulating immune‐inflammatory responses. Although the specific mechanism of this synergistic effect requires further investigation, our data preliminarily support that the combined supplementation of vitamin D and calcium may provide a better intervention strategy for sarcopenia.

Sarcopenia and frailty have common pathways, both being considered as strong predictors of disability (Nascimento et al. 2019). Identifying biomarkers that can be used for early intervention may be crucial to early prevention of disability. CXCL10 is functionally categorized as an inflammatory chemokine and may have important roles in leukocyte homing to inflamed tissues and in the perpetuation of inflammation, and thus, may importantly contribute to tissue damage (Lee et al. 2009). CXCL10, CX3CL1, and IL‐6 were identified as the core panel of frailty biomarkers (Cardoso et al. 2018). Our study showed that four dairy products could all reduce the levels of CXCL10 and IL‐6. We next used a multi‐omics study to explore metabolic and gut microbiota biomarkers in depth. After four dairy interventions, we found that the biomarker Leuconostoc was increased. Three goat dairy interventions could also lead to the biomarkers Acinetobacter, Lactococcus, and *Acinetobacter guillouiae* rising. Notably, goat low‐fat milk and fortified vitamin D and calcium low‐fat milk intake could increase the biomarker *Staphylococcus sciuri* abundance. Therefore, our study suggested that dairy interventions may have a role in the prevention of sarcopenia, frailty, and disability through affecting these targets.

Researchers are increasingly focusing their interests on the possible involvement of gut microbiota in the pathophysiology of sarcopenia. Alterations in the gut microbiota composition could in fact promote chronic inflammation and anabolic resistance, ultimately conditioning reduced muscle size, impaired muscle function, and adverse clinical outcomes (Grosicki et al. 2018). Our study showed an association between dairy intake and specific taxa (Grosicki et al. 2018; Li et al. 2024). Leuconostoc enriched in all dairy groups was proved to have the potential to be novel lactic acid bacteria (Chen et al. 2020). Lactococcus as another lactic acid bacteria was enriched in all goat dairy groups. Several studies have previously investigated that Lactococcus cremoris subsp. counteracted muscle atrophy by reduction in the levels of inflammatory factors (Aoi et al. 2022). Acinetobacter enriched in all goat dairy groups has been previously shown to be enriched in menopausal healthy women (Liu et al. 2022). These results suggest that goat milk may prevent sarcopenia partially by regulating intestinal flora.

The microbiota could be pathophysiologically involved in the onset of osteosarcopenic obesity (Hénique et al. 2015). In this study, we found that the abundances of specific species were associated with reduced inflammation in the body, such as IL‐1β, IL‐6, and TNF‐α. A higher dairy‐microbial score may indicate a greater ability to lower fat weight and suggest that gut microbiota may contribute to the variation in fat. In previous studies, dairy‐microbial scores were found to be negatively associated with blood triglycerides (Shuai et al. 2021). Interestingly, results from our correlation analysis lead to a hypothesis that gut microbiome may mediate the association of dairy intake with muscle synthesis. Our data suggested that leucine enriched by GFM was inversely associated with dairy‐microbial score. Previous studies reported that leucine administration increased muscle mass, strength, and physical function in patients with sarcopenia (Yoshimura et al. 2019; Martínez‐Arnau et al. 2020). These findings suggest that consumption of dairy foods, especially goat fortified vitamin D and calcium low‐fat milk, may contribute to the prevention of sarcopenia. Correlation analysis between metabolomics and intestinal flora function showed that dairy‐related gut microbiota was associated with the host circulating metabolomics profile, suggesting the beneficial association of dairy intake with sarcopenia (Qi et al. 2025).

Nevertheless, several caveats are important to note. The limitations of animal experiments for clinical application must be overcome, and an association between human gut microbiota composition and muscle mass has not been demonstrated yet. Furthermore, bioinformatic approaches suffer from intrinsic limitations, and the causal link of gut microbiota and sarcopenia remains uncertain.

## 5 Conclusion

This study demonstrates that different types of dairy products have distinct effects on muscle metabolism, autophagy, inflammation, and gut microbiota in sarcopenia. Goat milk, particularly its low‐fat and vitamin D/calcium‐fortified variants, showed greater benefits in promoting muscle regeneration and reducing inflammation compared to bovine milk, highlighting the importance of dairy composition. Additionally, dairy fat content influenced muscle metabolic pathways, with low‐fat formulations more effectively activating the PI3K/Akt/mTOR pathway and enhancing autophagy. Gut microbiota modulation further supported the role of dairy in muscle health, suggesting potential microbiome‐mediated mechanisms. These findings provide experimental evidence for optimizing dairy‐based nutritional strategies in sarcopenia prevention and management.

## Author Contributions

**Meng Sun:** conceptualization (equal), data curation (equal), formal analysis (equal), investigation (equal), methodology (equal), project administration (equal), software (lead), validation (equal), visualization (lead), writing – original draft (equal), writing – review and editing (equal). **Tong Wu:** conceptualization (lead), data curation (equal), formal analysis (lead), funding acquisition (equal), methodology (equal), project administration (equal), software (equal), validation (equal). **Ruoyu Wang:** methodology (equal), software (equal), validation (equal), writing – review and editing (equal). **Yuxin Ma:** formal analysis (equal), investigation (equal), resources (equal), visualization (equal). **Yaxin Han:** data curation (equal), software (equal), visualization (equal). **Yanmei Hou:** conceptualization (equal), project administration (equal), supervision (equal), validation (equal). **Zhaofeng Zhang:** conceptualization (equal), funding acquisition (equal), project administration (equal), resources (equal), supervision (lead), validation (equal), writing – review and editing (equal).

## Ethics Statement

This study was approved by the Biomedical Research Ethics Committee of Peking University (LA2022011).

## Conflicts of Interest

The authors declare no conflicts of interest.

## Supporting information


Data S1.

## Data Availability

The datasets presented in this study can be found in online repositories. The names of the repository/repositories and accession number(s) can be found below: https://www.ncbi.nlm.nih.gov; PRJNA 946942.
