# Supplementary material for: Four Dairy Products Mitigates Sarcopenia in Mice by Modulating Muscle Inflammation, Autophagy, and Protein Degradation
Source: Food Sci Nutr. 2025 Jul 14;13(7):e70540. doi: 10.1002/fsn3.70540 (PMC12256989; doi:10.1002/fsn3.70540)
Supplement: Supplementary file 1 — Data S1. [file FSN3-13-e70540-s001.docx]

## Supplementary Materials

**Supplementary Figure 1:** The animal experimental design.
**Supplementary Figure 2:** LEfSe significant differences in gut microbial abundance at the genus level and the species level of sarcopenic mice treated for 8 weeks with goat and bovine milk. (a) The genus level. (b) The species level. LDA score＞2.
**Supplementary Figure 3:** Heatmap of the correlation coefficients between metabolite types and gut microbiome functional pathways. The coloring indicates the direction of association (red: positive; blue: negative). *: *p* value＜.05; **: *p* value＜.01; ***: *p* value＜.001.
**Supplementary Table S1:** Detailed information on differential microbiota at the genus level in the SOP group compared with other groups.
**Supplementary Table S2:** Detailed information on differential microbiota at the species level in the SOP group compared with other groups.
**Supplementary Table S3:** Detailed information on potential metabolic biomarkers in the SOP group compared with other groups.

**
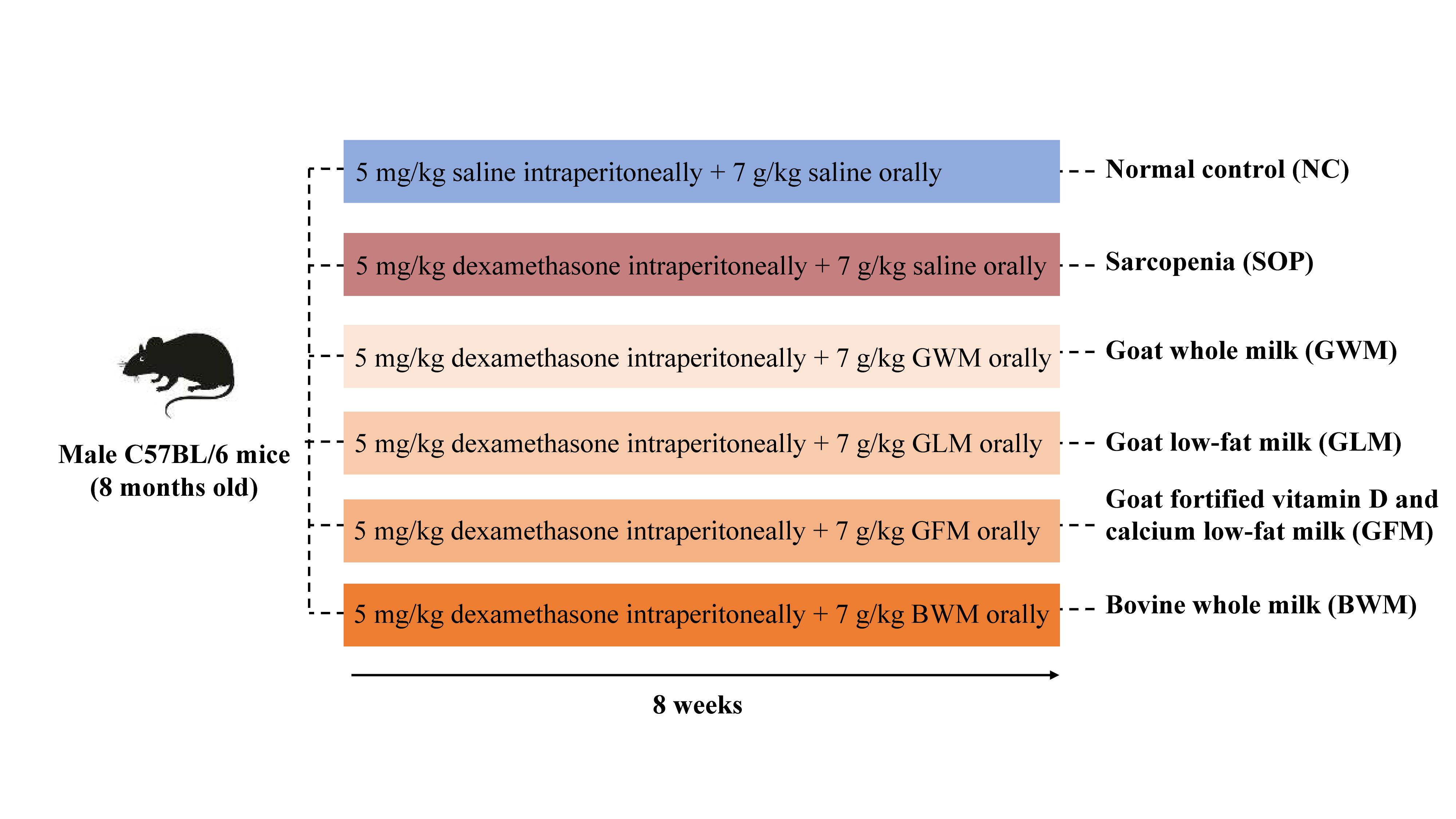
**

Supplementary Figure 1: The animal experimental design.


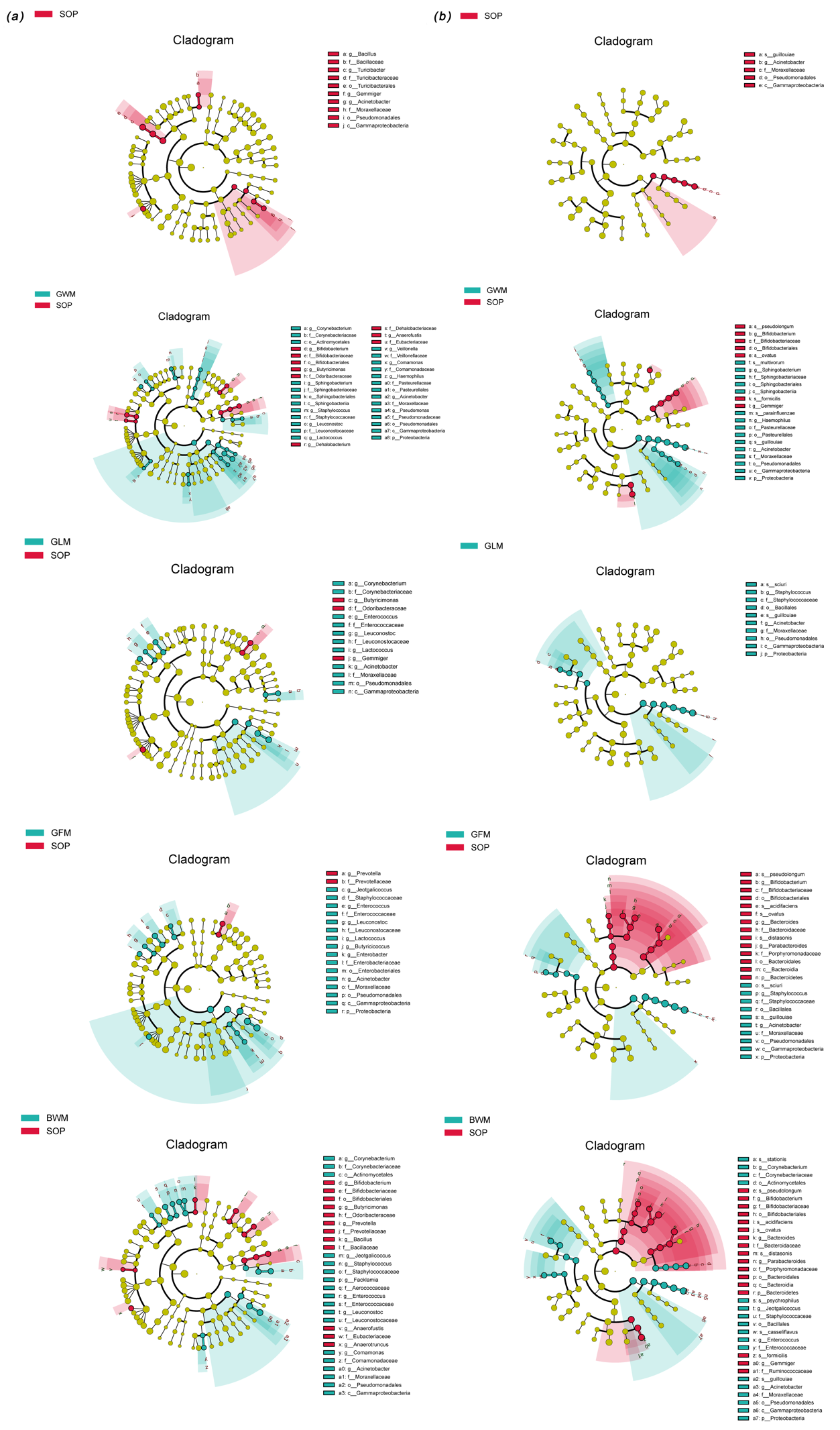


Supplementary Figure 2: LEfSe significant differences in gut microbial abundance at the genus level and the species level of sarcopenic mice treated for 8 weeks with goat and bovine milk. (a) The genus level. (b) The species level. LDA score＞2.


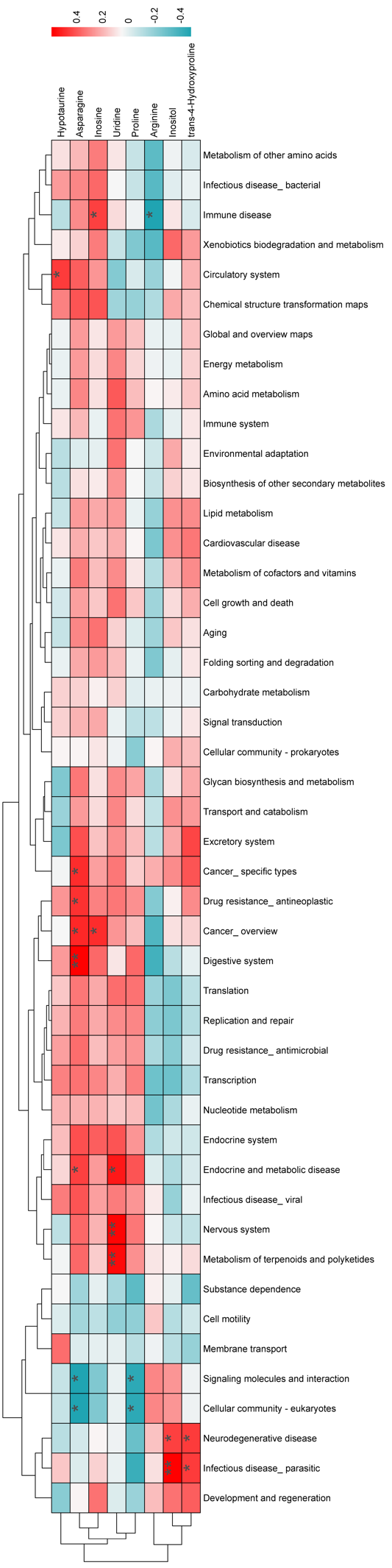


Supplementary Figure 3: Heatmap of the correlation coefficients between metabolite types and gut microbiome functional pathways. The coloring indicates the direction of association (red: positive; blue: negative). *: p value＜.05; **: p value＜.01; ***: p value＜.001.

Supplementary Table S1. Detailed information on differential microbiota at the genus level in the SOP group compared with other groups.

| Differential microbiota | LDA | *P*-vlaue Adjusted | Group of dominant bacterium | |
| --- | --- | --- | --- | --- |
| **NC group vs. SOP group (4)** | | | |  |
| Turicibacterales | 4.228 | 0.0472 | SOP | |
| Clostridiales | 3.6795 | 0.0283 | SOP | |
| Pseudomonadales | 3.6698 | 0.0163 | SOP | |
| Bacillales | 3.6666 | 0.0472 | SOP | |
|  |  |  |  | |
| **SOP group vs. GWM group (14)** | | | | |
| Bifidobacterium | 3.9328 | 0.0283 | SOP | |
| Dehalobacterium | 3.5079 | 0.0283 | SOP | |
| Butyricimonas | 3.2986 | 0.0090 | SOP | |
| Anaerofustis | 3.1274 | 0.0283 | SOP | |
| Acinetobacter | 4.3677 | 0.0090 | GWM | |
| Staphylococcus | 3.0623 | 0.0278 | GWM | |
| Sphingobacterium | 3.0314 | 0.0264 | GWM | |
| Veillonella | 3.0217 | 0.0343 | GWM | |
| Lactococcus | 3.0209 | 0.0283 | GWM | |
| Comamonas | 3.0032 | 0.0163 | GWM | |
| Haemophilus | 2.9989 | 0.0082 | GWM | |
| Leuconostoc | 2.9568 | 0.0082 | GWM | |
| Corynebacterium | 2.8790 | 0.0465 | GWM | |
| Pseudomonas | 2.7829 | 0.0465 | GWM | |
|  |  |  |  | |
| **SOP group vs. GLM group (6)** | | | | |
| Butyricimonas | 3.4845 | 0.0090 | SOP | |
| Acinetobacter | 3.9402 | 0.0283 | GLM | |
| Enterococcus | 3.3430 | 0.0283 | GLM | |
| Lactococcus | 3.2635 | 0.0283 | GLM | |
| Corynebacterium | 3.2527 | 0.0278 | GLM | |
| Leuconostoc | 3.2378 | 0.0082 | GLM | |
|  |  |  |  | |
| **SOP group vs. GFM group (8)** | | | | |
| Prevotella | 3.8810 | 0.0163 | SOP | |
| Acinetobacter | 4.7682 | 0.0090 | GFM | |
| Enterobacter | 4.1233 | 0.0264 | GFM | |
| Lactococcus | 4.0557 | 0.0090 | GFM | |
| Enterococcus | 3.8664 | 0.0090 | GFM | |
| Leuconostoc | 3.7390 | 0.0082 | GFM | |
| Jeotgalicoccus | 3.7298 | 0.0278 | GFM | |
| Butyricicoccus | 3.5945 | 0.0472 | GFM | |
|  |  |  |  | |
| **SOP group vs. BWM group (13)** | | | | |
| Bifidobacterium | 3.8673 | 0.0472 | SOP | |
| Anaerofustis | 3.5482 | 0.0283 | SOP | |
| Butyricimonas | 3.4828 | 0.0088 | SOP | |
| Prevotella | 3.3222 | 0.0283 | SOP | |
| Anaerotruncus | 3.2950 | 0.0472 | SOP | |
| Bacillus | 3.2687 | 0.0090 | SOP | |
| Corynebacterium | 3.8020 | 0.0088 | BWM | |
| Enterococcus | 3.7550 | 0.0472 | BWM | |
| Staphylococcus | 3.5470 | 0.0465 | BWM | |
| Comamonas | 3.4692 | 0.0090 | BWM | |
| Jeotgalicoccus | 3.4142 | 0.0160 | BWM | |
| Leuconostoc | 3.2424 | 0.0264 | BWM | |
| Facklamia | 3.1993 | 0.0236 | BWM | |

Supplementary Table S2. Detailed information on differential microbiota at the species level in the SOP group compared with other groups.

| Differential microbiota | LDA | *P*-vlaue Adjusted | Group of dominant bacterium | |
| --- | --- | --- | --- | --- |
| **NC group vs. SOP group (1)** | | | |  |
| Acinetobacter guillouiae | 4.2761 | 0.0090 | SOP | |
|  |  |  |  | |
| **SOP group vs. GWM group (6)** | | | | |
| Bifidobacterium pseudolongum | 4.9148 | 0.0283 | SOP | |
| Gemmiger formicilis | 4.6892 | 0.0283 | SOP | |
| Bacteroides ovatus | 4.6307 | 0.0283 | SOP | |
| Acinetobacter guillouiae | 5.1735 | 0.0090 | GWM | |
| Haemophilus parainfluenzae | 4.3546 | 0.0150 | GWM | |
| Sphingobacterium multivorum | 4.2909 | 0.0445 | GWM | |
|  |  |  |  | |
| **SOP group vs. GLM group (2)** | | | | |
| Acinetobacter guillouiae | 4.9687 | 0.0090 | GLM | |
| Staphylococcus sciuri | 4.5683 | 0.0150 | GLM | |
|  |  |  |  | |
| **SOP group vs. GFM group (5)** | | | | |
| Bacteroides ovatus | 5.0232 | 0.0090 | SOP | |
| Bifidobacterium pseudolongum | 4.9137 | 0.0283 | SOP | |
| Bacteroides acidifaciens | 4.8482 | 0.0283 | SOP | |
| Parabacteroides distasonis | 4.3530 | 0.0283 | SOP | |
| Acinetobacter guillouiae | 5.4058 | 0.0090 | GFM | |
| Staphylococcus sciuri | 4.5771 | 0.0445 | GFM | |
|  |  |  |  | |
| **SOP group vs. BWM group (9)** | | | | |
| Bifidobacterium pseudolongum | 4.8632 | 0.0163 | SOP | |
| Bacteroides acidifaciens | 4.8265 | 0.0472 | SOP | |
| Gemmiger formicilis | 4.7164 | 0.0283 | SOP | |
| Bacteroides ovatus | 4.3843 | 0.0283 | SOP | |
| Parabacteroides distasonis | 4.1842 | 0.0472 | SOP | |
| Acinetobacter guillouiae | 5.1418 | 0.0090 | BWM | |
| Corynebacterium stationis | 4.7333 | 0.0264 | BWM | |
| Jeotgalicoccus psychrophilus | 4.4705 | 0.0278 | BWM | |
| Enterococcus casseliflavus | 4.3680 | 0.0343 | BWM | |

Supplementary Table S3. Detailed information on potential metabolic biomarkers in the SOP group compared with other groups.

| Name of metabolite | Class | VIP | *P*-vlaue | Higher level group |
| --- | --- | --- | --- | --- |
| **NC group vs. SOP group (8)** | | | | |
| Leucine | Amino Acids | 1.9226 | 0.0353 | NC |
| Isoleucine | Amino Acids | 1.8378 | 0.0398 | SOP |
| Valine | Amino Acids | 1.9206 | 0.0363 | SOP |
| Pantothenic acid | Vitamins | 2.1696 | 0.0293 | NC |
| Asparagine | Amino Acids | 1.5760 | 0.0353 | NC |
| Tryptophan | Amino Acids | 1.5956 | 0.0217 | SOP |
| Phenylalanine | Amino Acids | 1.6860 | 0.0476 | SOP |
| Hypotaurine | Organic Acids | 1.5403 | 0.0187 | SOP |
|  |  |  |  |  |
| **SOP group vs. GWM group (2)** |  |  |  |  |
| Inosine | Nucleotides | 2.0137 | 0.0317 | GWM |
| Asparagine | Amino Acids | 2.4691 | 0.0298 | GWM |
|  | | | | |
| **SOP group vs. GLM group (4)** | | | | |
| Methionine | Amino Acids | 1.9734 | 0.0403 | GLM |
| Oleic acid | Fatty Acids | 1.9114 | 0.0358 | GLM |
| Pantothenic acid | Vitamins | 1.9654 | 0.0479 | GLM |
| Arginine | Amino Acids | 2.1707 | 0.0143 | GLM |
|  |  |  |  |  |
| **SOP group vs. GFM group (5)** | | | | |
| Valine | Amino Acids | 2.1362 | 0.0159 | SOP |
| Leucine | Amino Acids | 1.8906 | 0.0159 | GFM |
| Uridine | Nucleotides | 1.7877 | 0.0500 | GFM |
| Inositol | Alcohols | 2.0220 | 0.0150 | GFM |
| Glycolic acid | Organic Acids | 1.8059 | 0.0415 | GFM |
|  |  |  |  |  |
| **SOP group vs. BWM group (6)** |  |  |  |  |
| Oleic acid | Fatty Acids | 2.3689 | 0.0275 | BWM |
| Glutamine | Amino Acids | 2.1083 | 0.0336 | BWM |
| Arginine | Amino Acids | 2.4050 | 0.0076 | BWM |
| trans-4-Hydroxyproline | Amino Acids | 2.2251 | 0.0294 | BWM |
| Putrescine | Amines | 1.7294 | 0.0079 | BWM |
| Proline | Amino Acids | 2.1686 | 0.0367 | SOP |
|  |  |  |  |  |
